# The use of relative incidence ratios in self-controlled case series studies: an overview

**DOI:** 10.1186/s12874-016-0225-0

**Published:** 2016-09-23

**Authors:** Steven Hawken, Beth K. Potter, Julian Little, Eric I. Benchimol, Salah Mahmud, Robin Ducharme, Kumanan Wilson

**Affiliations:** 1 Clinical Epidemiology Program, Ottawa Hospital Research Institute, 725 Parkdale Ave, Ottawa, ON K1Y 4E9 Canada; 2 School of Epidemiology, Public Health and Preventive Medicine, University of Ottawa, 451 Smyth Road, Ottawa, ON K1H 8 M5 Canada; 3 Institute for Clinical Evaluative Sciences Ottawa, Box 684, 1053 Carling Ave., Admin Services Bldg. Rm 1009, Ottawa, ON K1Y4E9 Canada; 4 Division of Gastroenterology, Hepatology and Nutrition, Children’s Hospital of Eastern Ontario and Department of Pediatrics, University of Ottawa, 401 Smyth Road, Ottawa, ON K1H 8L1 Canada; 5 Department of Medicine, 727 McDermot Ave., Winnipeg, MB R3E 3P5 Canada

**Keywords:** Epidemiologic research design, Self-controlled case series, Vaccination, Vaccine safety

## Abstract

**Background:**

The self-controlled case series (SCCS) is a useful design for investigating associations between outcomes and transient exposures. The SCCS design controls for all fixed covariates, but effect modification can still occur. This can be evaluated by including interaction terms in the model which, when exponentiated, can be interpreted as a relative incidence ratio (RIR): the change in relative incidence (RI) for a unit change in an effect modifier.

**Methods:**

We conducted a scoping review to investigate the use of RIRs in published primary SCCS studies, and conducted a case-study in one of our own primary SCCS studies to illustrate the use of RIRs within an SCCS analysis to investigate subgroup effects in the context of comparing whole cell (wcp) and acellular (acp) pertussis vaccines. Using this case study, we also illustrated the potential utility of RIRs in addressing the healthy vaccinee effect (HVE) in vaccine safety surveillance studies.

**Results:**

Our scoping review identified 122 primary studies reporting an SCCS analysis. Of these, 24 described the use of interaction terms to test for effect modification. 21 of 24 studies reported stratum specific RIs, 22 of 24 reported the *p*-value for interaction, and less than half (10 of 24) reported the estimate of the interaction term/RIR, the stratum specific RIs and interaction *p*-values. Our case-study demonstrated that there was a nearly two-fold greater RI of ER visits and admissions following wcp vaccination relative to acp vaccination (RIR = 1.82, 95 % CI 1.64–2.01), where RI estimates in each subgroup were clearly impacted by a strong healthy vaccinee effect.

**Conclusions:**

We demonstrated in our scoping review that calculating RIRs is not a widely utilized strategy. We showed that calculating RIRs across time periods is useful for the detection of relative changes in adverse event rates that might otherwise be missed due to the HVE. Many published studies of vaccine-associated adverse events could have missed/underestimated important safety signals masked by the HVE. With further development, our application of RIRs could be an important tool to address the HVE, particularly in the context of self-controlled study designs.

## Key messages

- The self-controlled case series design (SCCS) is a case-only design, and as such the within-subject SCCS analysis controls for all fixed baseline covariates and has become a method of choice for studies of adverse events following vaccination.
- Despite adjustment for baseline covariates, effect modification can still occur in SCCS analyses and can be tested by including interaction terms in the SCCS model. When exponentiated, these interaction terms can be interpreted as relative incidence ratios (RIR).
- In this paper we present the results of our scoping review investigating the use of RIRs in published SCCS analyses, and also a case-study using one of our primary SCCS studies applying RIRs to investigate comparative subgroup effects, and as a mechanism to improve the detection and quantification of safety signals in the presence of the healthy vaccinee effect (HVE) in vaccine safety surveillance studies.
- Many published studies of adverse events immediately following vaccinating using SCCS could have underestimated or failed to detect important safety signals by not recognizing and addressing the impact of the HVE.

## Background

Post-marketing surveillance is important for ongoing evaluation of the safety of vaccines, and is typically based on observational data, for which conventional study designs (eg. case-control, cohort) are particularly vulnerable to confounding. This is because many factors that are associated with avoidance or delay of vaccination are also associated with the health outcomes of interest [1–4].

The self-controlled case series design (SCCS) was developed to address a number of challenges associated with studying the association between adverse health outcomes and transient exposures, such as vaccination, in observational data. The SCCS is a case-only design where inference is based on disease cases and their exposures, in which each individual serves as his or her own control, implicitly adjusting for all fixed covariates (eg. sex, socio-economic status) [5–7]. The SCCS is fit with a conditional Poisson regression model, for which general use SAS macros and R functions have been made available by authors of the SCCS methodology in addition to extensive reference material and examples (http://statistics.open.ac.uk/sccs) [6].

The fitted SCCS conditional Poisson model provides estimates of relative incidence (RI) of adverse events, comparing incidence in exposed periods (eg. immediately following a vaccination) to unexposed periods, within individuals. The SCCS has important advantages: 1) it addresses confounding resulting from differences between the vaccinated and unvaccinated individuals in non-randomized study settings; 2) traditional cohort and case-control designs may not be feasible for studying vaccines with coverage approaching 100 % as it would be difficult to recruit unvaccinated controls; and 3) safety surveillance systems typically only collect data for individuals who reported an adverse event thought to be related to vaccination.

Despite the built-in control of time-invariant confounders in an SCCS model, it is possible that effect modification (interaction) exists such that the magnitude and/or direction of the RI differs according to one or more (fixed) covariates (e.g. age, sex, socio-demographic factors, co-morbidities). Within the framework of an SCCS model it is possible to address interactions between exposure (e.g. vaccination) and one or more fixed covariates with respect to the outcome of interest by including interaction terms [6]. The exponentiated parameter estimate for the interaction term can be interpreted as a “relative incidence ratio” (RIR) as it is exactly equivalent to the ratio of the RI in one group compared to the RI in the designated reference group (if the interacting variable is categorical) or the change in the relative incidence given a one unit increase in the covariate (if the interacting variable is continuous). We have previously published a number of studies comparing RIs for adverse events following immunization (AEFI) among important subpopulations using RIRs. We have reported that rates of ER visits and admissions vary according to: quintiles of birth weight [8], birth order [9], quintiles of neighborhood income [10], sex [11] and gestational age at birth (prematurity) [12]. We have also used RIRs to compare the safety of influenza vaccination in patients with inflammatory bowel disease to that in healthy controls [13].

**Fig. 1:**
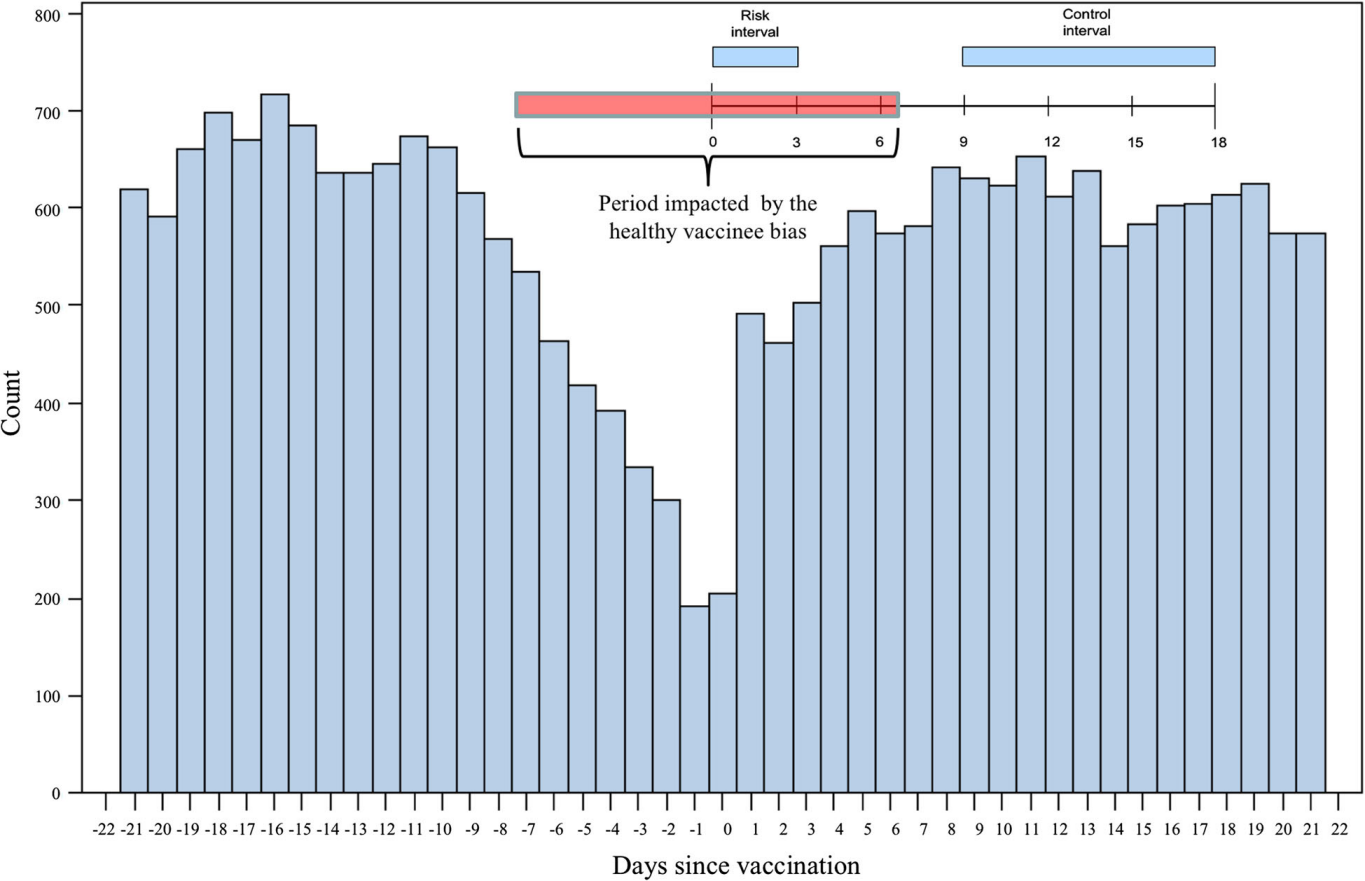
Frequency of ER visits and hospital admissions in the 3 weeks before and after vaccination (DTaP-IPV-Hib at 6 months of age): Illustration of the impact of the healthy vaccinee effect: Data from Ontario, Canada [22]. Count = number of combined endpoints of emergency room visit, hospitalization and death. Days since vaccination = number of days before or after vaccination, day 0 being the day of vaccination

Despite its numerous strengths, the SCCS is not immune to a critical but perhaps under-recognized phenomenon that can influence the interpretation of an SCCS analysis: the healthy vaccinee effect (HVE) [14–16]. If an individual has been ill, recently hospitalized, or otherwise unwell, vaccination may be deferred by the provider/patient/parent/guardian until the health of the individual improves. This is especially true for vaccinations in early infancy. For this reason, when observing the health status of an individual with a completed vaccination, this individual is more likely to be in a healthy state immediately before and after their vaccination. The HVE has the effect of reducing event rates in the immediate pre- and post-vaccination periods. The impact of the HVE is particularly evident in studies that utilize non-specific outcome measures, for example health service utilization (hospital admissions, ER visits, physician visits), as a metric for evaluating AEFIs. Figure 1 illustrates the healthy vaccinee effect for ER visits and admissions relative to date of vaccination for routine pediatric vaccination at age 6 months in Ontario, Canada.

The impact of the HVE may be much harder to quantify for less common events such as convulsions, and nearly impossible for extremely rare events (e.g. encephalitis, hypotonic hypo-responsive episodes (HHE)). For these rare and more serious outcomes, the HVE may also not have enough of an impact to be relevant, but there is not enough data available to confirm this. It is possible that studies reporting no increased risk of adverse events following vaccination may in fact have missed clinically important safety signals that were distorted by the HVE. Although the HVE is acknowledged in the literature, its potential impact on the detection of adverse events in the first few days following a completed vaccination is not as well recognized.

Our study sought to: 1) Investigate the use of RIRs among all published primary SCCS studies through a comprehensive scoping review, and 2) Present our case-study applying relative incidence ratios (RIRs) in the analysis of one of our own primary SCCS studies of adverse events following vaccination to investigate subgroup effects in the context of comparing whole cell (wcp) and acellular (acp) pertussis vaccines. Using this case study, we also sought to demonstrate the potential utility of RIRs in addressing the healthy vaccinee effect (HVE) that could mask important safety signals in many vaccine safety surveillance studies.

## Methods

### Scoping review of use of RIRs in primary SCCS studies

We searched the PubMed and Scopus databases for all papers published between January 1st, 1995 and April 30th, 2014 with the keywords “self-controlled case series” OR “self-controlled risk interval” OR “self-controlled cohort”, and also all papers that cited any of the main methods papers describing the SCCS [6, 7, 17–21]. The titles and abstracts were then reviewed and all studies reporting an original analysis of observational data using the SCCS methodology were retained for full review. The full texts of the retained manuscripts were then reviewed to determine if interaction tests were performed in the SCCS model, how they were applied and how the results were reported, as well as searching the references cited in the reviewed papers for additional studies that may have been missed. Detailed information about each study was extracted and reported in the study results.

### Case-study of the application of relative incidence ratios (RIRs) in an SCCS analysis

The diphtheria, tetanus, acellular pertussis, inactivated polio and Haemophilus influenzae b (DTaP-IPV-Hib) component vaccine is given at 2, 4 and 6 months of age in Ontario, Canada. For component vaccines, adverse reactions typically occur immediately following a vaccination (0–72 h post-injection) because they contain no live replicating virus. Under these conditions, the risk period overlaps with the period in which reduced rates of adverse events are observed due to the HVE. Therefore, when observing the incidence of adverse events in the most likely risk period (from 0 to 72 h post-injection for DTaP-IPV-Hib) versus a control period farther removed from vaccination, the HVE may mask increased adverse event risk associated with vaccine administration.

We have previously conducted a study of emergency room (ER) visits and admissions following DTaP-IPV-Hib vaccine recommended at ages 2, 4 and 6 months in which we found no evidence of increased risk of events in the first 72 h following vaccination (compared to the control period: days 9 to 18), but we observed a strong HVE, which is described by Fig. 1 [22]. In the studies we have conducted using the SCCS, our implementation of the design was somewhat atypical in that we defined very short control periods (e.g. 9 days) and only included exposure time in the post-vaccination period [8–12, 22–24]. Our rationale for the selected control periods was two-fold. Firstly, when studying vaccinations in early infancy, background rates of ER visits and hospitalizations change very rapidly, especially in the first few months following birth. Therefore, careful age stratification is required to control for changing background event rates when longer control intervals are used, which would further complicate the analysis. Secondly, as vaccinations in first year after birth are closely spaced (e.g. 2, 4, 6 and 12 months), tight control intervals were required in order to investigate associations involving individual vaccinations so that the control intervals did not overlap with risk periods associated with subsequent vaccinations. Although we used an atypical implementation of unexposed control periods in our case-study, the methods applied in our case study are broadly applicable to SCCS models in general.

## Results

### Scoping review of use of RIRs in primary SCCS studies

Our electronic database search returned 334 articles. Titles and abstracts were reviewed to eliminate duplicates, and to identify those studies that reported an original analysis of observational data using the SCCS methodology, leaving 122 studies. After retrieval and full text review of the 122 remaining studies, 24 described the use of interaction terms in the SCCS model to test for effect modification. No additional studies were identified through references listed by the reviewed papers. Details of the 24 included studies are given in Table 1. For the final subset of qualifying manuscripts, 10 of 24 studies reported the estimate of the interaction term/RIR in addition to the stratum specific relative incidence estimates and interaction *p*-values. Twenty-one of 24 studies reported the stratum specific RIs, and 22 of 24 reported the *p*-value for the test for interaction. Two of 24 studies reported no details of the interaction tests, other than that they were performed, and did not achieve statistical significance (Table 1).

**Table 1:** Studies that reported testing for interactions, and/or comparing subgroups in an SCCS modeling context

Study ^a^	Exposure	Outcomes	Interaction Tested	Estimates/RIR/int. *p*-value reported
Wilson et al. [11]	12 month MMR vaccination	ER visits + admissions	Sex	yes/yes/yes
Kwong et al. [26]	Influenza illness and influenza immunization	Guillain-Barré syndrome	Age, sex, month of vaccination	yes/no/yes
Benchimol et al. [13]	Influenza vaccination	ER visits + admissions + physician visits IBD flares	IBD versus healthy controls	yes/yes/yes
Wilson et al. [10]	2, 4, 6 (DTaP) and 12 month (MMR) vaccination	ER visits + admissions	SES (Neighborhood income quintiles)	yes/yes/yes
Hawken et al. [23]	Acellular/whole cell pertussis vaccine	ER visits + admissions	Whole cell (1994–1996) versus acellular pertussis vaccine (1998–2000)	yes/yes/yes
Wilson et al. [12]	2 month vaccination	ER visits + admissions	Preterm versus full term infants	yes/yes/yes
Wilson et al. [8]	2, 4, 6 and 12 month vaccination	ER visits + admissions	Quintiles of birthweight	yes/yes/yes
Connolly-Anderson et al. [27]	Hemorrhagic fever with renal syndrome	Acute myocardial infarction and stroke	Sex	yes/no/yes
Langan et al. [28]	Herpes Zoster infection	Stroke	Antiviral Therapy	yes/no/yes
Dodd et al. [29]	H1N1 vaccination	Guillain-Barré syndrome	Age, sex, adjuvanted vs. non-adjuvanted vaccine, concomitant seasonal flu vaccine	yes/no/yes
Butt et al. [30]	Antihypertensives	Falls	Sex	yes/no/yes
Andrews et al. [31]	MMR vaccination	Thrombocytopenic purpura	Country	yes/no/yes
Tokars et al. [32]	H1N1 and seasonal influenza vaccination	Guillain-Barré syndrome	age, sex, vaccine type, received season flu vaccine, site	yes/no/yes
Pariente et al. [33]	Antipsychotic use	Myocardial infarction	Previous history of cardiovascular disease	no/no/no^b^
Warren-Gash et al. [34]	Influenza vaccination	Acute MI	Age group, sex, type of infarction, and history of vascular disease	yes/no/yes
Tse et al. [35]	Influenza vaccination	Febrile seizures	concomitant 13-valent pneumococcal conjugate vaccine (PCV13), age	yes/yes/yes
Gwini et al. [36]	Influenza vaccination	Acute myocardial infarction	Age, sex	yes/no/yes
Pattenden et al. [37]	Heat Exposure	Mortality	Ozone levels	yes/yes/yes
Andrews et al. [38]	Acellular pertussis/whole cell pertussis vaccine	Convulsions,	Whole cell period vs. acellular period	yes/yes/yes
Douglas et al. [39]	Thiazolidinediones	Fractures	Rosiglitazone versus pioglitazone	yes/no/yes
Miller et al. [40]	MMR	Convulsions and aseptic meningitis	Vaccine Manufacturer, Concomitant MCC vaccination vs. separate	yes/no/yes
Game et al. [41]	Initiation of dialysis	Foot ulceration	Haemodialysis vs. ambulatory peritoneal dialysis	no/no/no^a^
Miller et al. [42]	MMR vaccination	Gait Disturbance	doses of thimerisol containing vaccines by 4 months, \ mercury exposure intensity by 6 months	yes/no/yes
Sardinas et al. [43]	Oral polio vaccine	Intussusception	age	no/no/yes

^a^Studies reported above the gray dividing bar are studies published by the authors and their collaborators^b^Reported that interaction was tested and did not reach statistical significance

### Case-study of the application of RIRs in an SCCS analysis

Table 2 presents a comparison of relative incidence of ER visits and admissions observed among children in Ontario, Canada in the 3 days following the 2-month diphtheria-whole-cell-pertussis-tetanus (DPT) vaccination (risk period) vs. days 9 to 18 (control period). We compared the RI during the period 1994 to 1996 when the more reactogenic whole cell pertussis vaccine was being administered with the RI during the period from 1998 to 2000 when the less reactogenic diphtheria-tetanus-acellular pertussis vaccine (DTaP) was being administered. The RI during the whole cell period was 1.08 (95 % CI 1.02–1.15) and the RI during the acellular period was 0.60 (95 % CI 0.55–0.65). The RIR for the whole cell versus acellular period was calculated to be 1.82 (95 % CI 1.64–2.01). This provides evidence that the relative incidence of events was nearly two-fold higher in the whole-cell versus acellular vaccine usage periods.

**Table 2:** ER Visits and Admissions in the first 72 h following vaccination versus days 9 to 18 for the time period when whole cell pertussis vaccine was used compared to the time period when acellular pertussis vaccine was used [23]

	Risk events (first 72 h)	Control events (Day 9–18)	Relative incidence	95 % CI	Relative incidence ratio	95 % CI	*P*-value^a^
Apr 94–Mar96 (whole-cell)	1323	3663	1.08	1.02–1.15	1.82	1.64–2.01	*P* < 0.0001
Apr98–Mar00 (acellular)	697	3508	0.60	0.55–0.65	1 (ref)	-	-

^a^ *p*-value for interaction of time period (whole cell or acellular periods) with risk

Employing the parameter estimates generated from the fitted conditional Poisson model, the RI in each time period as well as the RIR comparing the whole cell versus acellular period can be expressed as:

$$ \begin{array}{c}\hfill \mathrm{R}\mathrm{I}\left(\mathrm{whole}\ \mathrm{cell}\ \mathrm{period}\right)= \exp \left(1\cdot {\beta}_{risk}+1\cdot {\beta}_{risk}\cdot 1\cdot {\beta}_{period}\right)=1.08\hfill \\ {}\hfill \mathrm{R}\mathrm{I}\left(\mathrm{acellular}\ \mathrm{period}\right)= \exp \left(1\cdot {\beta}_{risk}+1\cdot {\beta}_{risk}\cdot 0\cdot {\beta}_{period}\right)= \exp \left(1\cdot {\beta}_{risk}\right)=0.60\hfill \\ {}\hfill \mathrm{R}\mathrm{I}\mathrm{R}\ \left(\mathrm{whole}\ \mathrm{cell}\ \mathrm{v}\mathrm{s}.\ \mathrm{acellular}\ \mathrm{period}\right)= \exp \left(1\cdot {\beta}_{period}\right)=\frac{1.08}{0.60}=1.82\hfill \end{array} $$

where *β*_*risk*_ is the parameter estimate of log (RI) in the reference group (acellular period) and *β*_*period*_ is the parameter estimate of the interaction term of period (whole cell vs. acellular period) with risk. Since this is a dichotomous interaction variable, the *period* variable would take the value 1 for the whole cell period and 0 for the acellular period. For a categorical interaction with *m* categories, *period* would be a vector of (*m*-1) dummy variables indicating subgroup membership, or *period* may be a continuous variable. The main effect terms for the fixed covariates interacting with exposure cancel out (eg. biological sex does not change between an individual’s exposed and unexposed periods) leaving just the interaction terms. A likelihood ratio test is employed to compare the model with interaction terms included to the model that excludes these terms. This tests the hypothesis that the relative incidence of outcomes with respect to exposure (i.e. vaccination) depends on the value of the covariate involved in the interaction [6, 7].

**Fig. 2:**
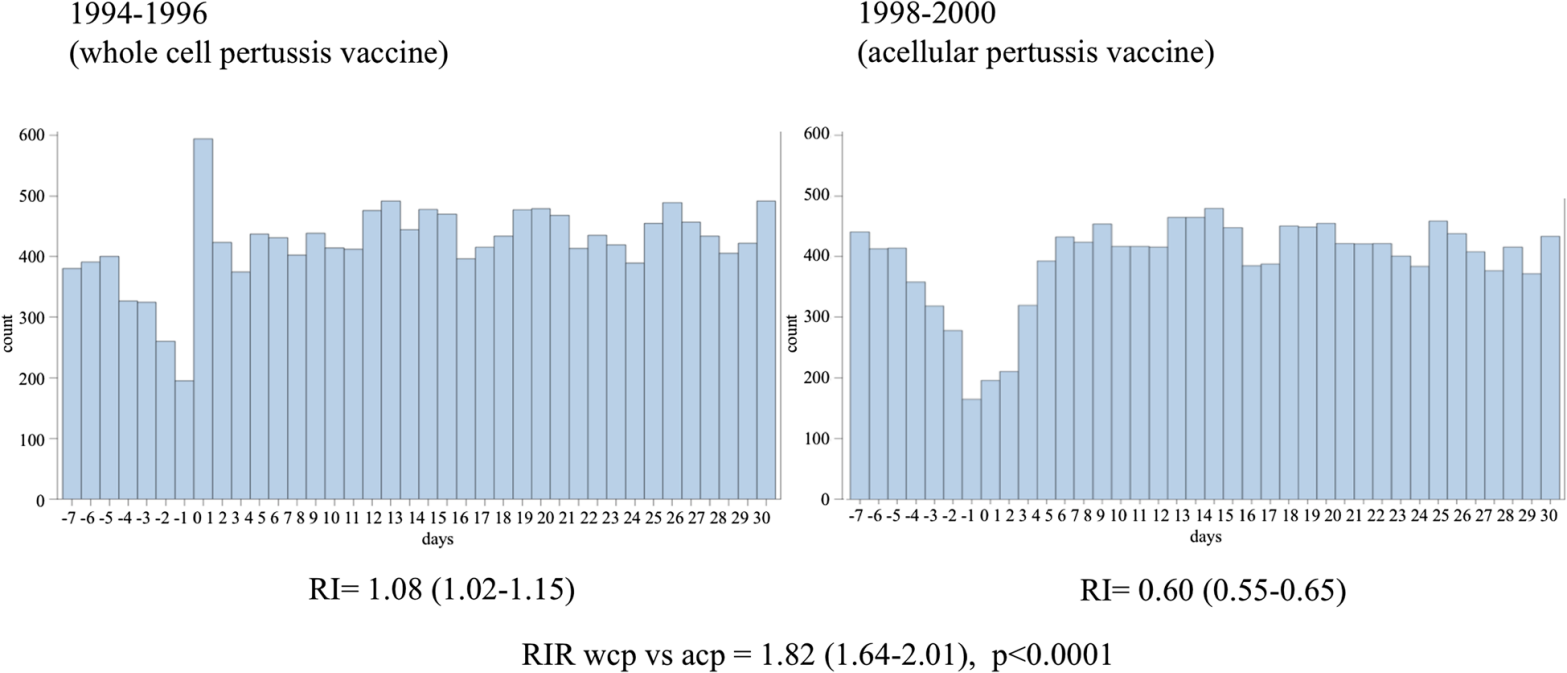
Emergency room visits and admissions before and after 2-month vaccination for whole cell pertussis combination vaccine (1994–1996) versus acellular pertussis vaccine (1998–2000). Count = number of combined endpoints of emergency room visit, hospitalization and death. Days = number of days before or after vaccination, day 0 being the day of vaccination

Visual inspection of the daily frequency of events relative to day of vaccination in both periods strongly suggest that the RI estimates in both the whole cell and acellular periods were impacted by the healthy vaccinee effect (Fig. 2). The effect is very similar in the week preceding vaccination in both the whole cell and the acellular pertussis periods, but in the days following the whole cell vaccinations (1994–1996), there is clearly a spike in events on the first day following vaccination which is largely washed out by the HVE (RI 1.08 = 95 % CI 1.02–1.15) but is still statistically significant with *p* < 0.0001 due to high statistical power. However, in the acellular period, there appears to be no spike following vaccination, but rather in the days following vaccination there is an approximate mirror image of the decrease in event rate seen before vaccination (RI = 0.60, 95 % CI 0.55–0.65). In both periods, the daily frequency of events is nearly halved by the day before vaccination with very similar relative incidence for the week before vaccination compared to the control period (days 9–18) (Fig. 2).

When we calculated the RIR for the whole cell versus acellular periods, the similar HVE in both periods is essentially cancelled out and the higher relative incidence of adverse events associated with the whole cell combination vaccine becomes clear (RIR = 1.82, 95 % CI 1.64–2.01) (Fig. 2). This suggests that RIRs provide a useful effect estimate that can be constructed in such a way to potentially overcome the healthy vaccinee effect to detect safety signals (or relative changes in safety signals) that might otherwise be missed.

## Discussion

In this manuscript, we presented the results of our scoping review of primary SCCS studies and the use of relative incidence ratios therein, as well as case-study from one of our primary SCCS studies providing a worked example of the utility, strengths and limitations of RIRs for describing subgroup effects as well as effect modification by continuous covariates.

In the context of vaccine safety surveillance, we are often interested in detecting increases or decreases in incidence of serious adverse event and changes in healthcare utilization patterns related to vaccine reactions following introduction of a new vaccine formulation, manufacturer, or other modification. In this case, interest may be focused on detecting changes in relative incidence over time or in important subgroups. Using relative incidence ratios (RIR), the change in relative incidence across time or physical subgroups of interest can be estimated and formally tested. For example, if a different formulation of a vaccine is introduced at a known point in time, then an SCCS model can be fit, with common risk and control periods across the entire population, but an interaction term is then included in the model, which estimates the RIR comparing the period after the new vaccine is introduced versus the period in which the old formulation was used.

In our case-study we have illustrated how, in some cases, the HVE can bias the observed relative incidence of adverse events that occur within the first few days following a vaccination. Calculating the RIR across groups to be compared (or in our case-study, time periods to be compared) similarly affected by the HVE would, in effect, cancel out the HVE and provide a potentially less biased estimate of the change in RI across the subgroups. Therefore, this could present a useful strategy for overcoming the healthy vaccinee effect, when relative changes in rates of adverse events across time periods, jurisdictions or subgroups of vaccines are of primary interest.

In the SCCS modeling context, RIRs are not afforded the same protection from confounding as the subgroup specific RI estimates, which is a potential limitation of our proposed applications of RIRs. For example, if we suspect that the relative incidence of adverse events depends on sex (i.e. females are more susceptible to adverse events following vaccination than males who are similarly exposed) we would test this hypothesis by including an interaction term between risk period and sex. This term, if statistically significant, provides evidence that sex is an effect modifier. However, since we would now be estimating an interaction effect across levels of a fixed baseline covariate, this could no longer be considered a within-individual effect estimate, and hence, any observed interaction effect could be the result of confounding. This issue can be addressed in the same way it is addressed in other modeling situations, by statistically controlling for other potential confounders in the SCCS model in order to assess whether the interaction effect of interest persists after statistical adjustment. This further adjustment is implemented by introducing additional interaction terms for the potential confounder(s) of interest and then observing whether the parameter estimate of the target effect modifier changes substantively. Stratified analysis is also a useful strategy to observe whether the interaction of interest is consistent across subgroups of known potential confounders of interest. These remedies afford less reassurance than the basic SCCS model, which provides main effect estimates that are implicitly controlled for all known and unknown fixed covariates. In generating RIR estimates we are limited to adjusting for known confounders, for which data are available. Vanderweele and Knol [25] point out that, if the point of the subgroup analysis is to identify vulnerable subsets of individuals for possible intervention, then confounding in interaction effects is of much less importance. If the aim is to make causal inference with respect to the source of the interaction, then the confounding would be important to account for [25].

## Conclusions

In this review we have demonstrated the potential utility of RIRs, which are based upon a test of interaction in the SCCS conditional Poisson model. We have discussed the strengths and limitations of RIRs for describing subgroup effects as well as effect modification by continuous covariates. We have also conducted a scoping review that demonstrated that very few primary SCCS studies are making use of RIRs to evaluate relative subgroup effects.

We have proposed that calculating RIRs across time periods (year over year for example) is very useful for detecting relative changes in rates of adverse events, which could constitute safety signals that might otherwise be missed due to the HVE. We emphasize many published studies of adverse events immediately following vaccination could have underestimated or failed to detect potentially important safety signals masked by the HVE. Our proposed application of RIRs could be an important tool in future studies to address the HVE. Further study is needed, including simulation studies and case studies in real data to assess the impact of different patterns of healthy vaccinee effect, the impact of increasing severity of confounding, as well as other violations of assumptions, on the reliability of inference using RIRs in post-marketing surveillance using the SCCS study design.

## Abbreviations

DTaP: Diphtheria-tetanus-acellular pertussis vaccine
DTaP-IPV-Hib: Diphtheria, tetanus, acellular pertussis, inactivated polio and Haemophilus influenzae b combination vaccine
DPT: Diphtheria-whole-cell pertussis tetanus vaccine
ER: Emergency room
HHE: Hypotonic hypo-responsive episodes
HVE: Healthy vaccinee effect
MMR: Measles mumps and rubella vaccine
RI: Relative incidence
RIR: Relative incidence ratio
SCCS: Self-controlled case series
